# Distinct transcriptome profiles reveal gene expression patterns during fruit development and maturation in five main cultivated species of pear (*Pyrus L.*)

**DOI:** 10.1038/srep28130

**Published:** 2016-06-16

**Authors:** Ming-Yue Zhang, Cheng Xue, Linlin Xu, Honghe Sun, Meng-Fan Qin, Shaoling Zhang, Jun Wu

**Affiliations:** 1 Centre of Pear Engineering Technology Research, State Key Laboratory of Crop Genetics and Germplasm Enhancement, Nanjing Agricultural University, Nanjing 210095, China; 2 Beijing Academy of Agriculture and Forestry Sciences, Key Laboratory of Biology and Genetic Improvement of Horticultural Crops (North China), Beijing, 100097, China

## Abstract

The transcriptomes of five pear cultivars, ‘Hosui’ (*P. pyrifolia*), ‘Yali’ (*P. bretschneideri*), ‘Kuerlexiangli’ (*P. sinkiangensis*), ‘Nanguoli’ (*P. ussuriensis*), and ‘Starkrimson’ (*P. communis*) were sequenced at seven key fruit developmental stages, from fruit setting to maturation and fruit senescence after harvesting. In total, 33,136 genes that could be mapped by reads, were analyzed. Most gene expression cluster models showed a steadily decreasing trend. Gene expression patterns had obvious differences according to maturity type, that is, post-ripening cultivars were still vigorous at maturity, and showed a higher proportion of up-regulated genes; non post-ripening cultivars had a gradually decreasing tendency during fruit maturation. Meanwhile, differentially expressed genes related to fruit quality and development, such as stone cells, sugar, acid and hormones, were identified. Co-expression analysis revealed that several ethylene synthesis genes and polyphenoloxidase-related genes interacted with each other directly, and an indirect relationship was reflected between ethylene synthesis genes and ethylene response genes. In addition, the highly diverse SNPs represented the great differences between oriental and occidental pears. Understanding how RNA-seq based gene-expression patterns and differential gene expression contribute to fruit quality allows us to build models for gene-expression for fruit development of *Pyrus* species.

Pear (*Pyrus* L.) belongs to the family of Rosaceae, with a history of cultivation dating back more than two thousand years in China^1^. It is well known that the pear originated in southwest of China, with diverse centers of evolution in China, Central Asia and Near Easter/Asia Minor^2^. Even though up to 22 pear species have been identified^3^, only five species are cultivated worldwide, four deriving from oriental countries, including *P. pyrifolia*, *P. bretschneideri*, *P. sinkiangensis* and *P. ussuriensis*, with only *P. communis* deriving from an occidental country. The geographical distribution and fruit genotype are diverse between the five pear species. In China, all five species have been cultivated around the country in appropriate cultivation areas. *P. pyrifolia* is cultivated in the south of China, *P. bretschneideri* in northern China, *P. ussuriensis* usually in the northeast region with extreme low temperatures, while *P. sinkiangensis* is only found in Xinjiang, and *P. communis* is cultivated in the north of China with cold climate, such as Beijing and Shandong province. For different cultivated species, typical maturity phenotypes exist between the five pear species. Generally speaking, fruit of *P. communis* and *P. ussuriensis* ripen after harvest, and their pears are usually eatable with good flavor and fruit quality after post harvest storage, while the other three cultivated species are eatable immediately when harvested at mature stage. In addition, different fruit traits are also found in the five species. The *P. communis* pears are gourd shaped, have soft and smooth flesh, with few stone cells^4^ and high sugar and acid content^5^, with sweet aroma. *P. ussuriensis* pears are usually high in volatile compounds^6^ and concentrated fruit flavor. On the contrary, the other oriental pears have crisp flesh, low aroma and flavor, and some of them have high sugar and low acid content^5^.

Until now, some physiological and molecular mechanism studies have been carried out to investigate different fruit development characteristics and fruit quality. For the fruit maturation process, fruit texture is affected by the *ACO* (1-aminocyclopropane-1-carboxylate oxidase) gene, and then *XTH* (Xyloglucan endotransglucosylase/hydrolase)-related genes can lead to cell wall disassembly and loosening^7^. ‘Bartlett’ occidental pears are prevented from ripening on the tree, but ethylene was able to stimulate fruit softening on the tree^8^ and endo-*PG* genes have an impact on many different maturation characteristics^8^,^9^. Microarrays were developed for pear to illustrate the absence of *cupin family protein*-related genes and two un-annotated genes in Japanese pear (*Pyrus pyrifolia*), which may induce post-ripening in pears^10^. Sugar is one of the most important factors for fruit quality, and provides the main energy for fruit metabolism. In *Pyrus* species, sucrose and fructose are the major soluble sugars, and overall sugar content varies greatly between different cultivars^5^. It was found that sucrose synthase and sucrose-phosphate synthase were highly correlated with sucrose levels in Japanese pear^11^. Soluble acid invertase accumulates hexoses and regulates sucrose-to-hexose ratio, and two invertase genes, *PsS*-*AIV1* and *PsS*-*AIV2*, have been cloned in pears, but are expressed differently during fruit development^12^. Lignified stone cells are a special characteristic of pears, which severely affect fruit quality. The stone cells have a mosaic pattern in the flesh of pear fruits, with a higher concentration of larger stone cells near the core, and smaller ones around the pericarp^13^. The ratio of G-type and S-type lignin and polymerization degree have been evaluated among various pear species^14^. Highly expressed gene families of *HCT, C3*′*H*, and *CCOMT* contribute to high accumulation of both G-lignin and S-lignin in ‘Dansuansuli’ fruit. However, there are few reports on all five cultivated pear species toward understanding the different molecular regulation mechanisms of fruit development and quality.

Next-generation RNA sequencing can sensitively and rapidly reflect gene expression with an enormous amount of data, and is an efficient method to explore fruit development characteristics. Besides pear, many Rosaceae family fruits are popular, such as apple, peach, berry, strawberry, plum, loquat, and apricot. The whole genomes of apple^15^, strawberry^16^, plum^17^, pear^18^ and peach^19^ have been sequenced, making possible differentially expressed gene analysis based on the reference genome to reveal different genotypes. In a previous study, transcriptome sequencing of apple helped to find the Gypsy-like retrotransposon in the bud mutant, affecting internode length^20^. Peach^21^ and berry^22^ have also been analyzed by high-throughout sequencing technology to reveal internal mechanisms of fruit development and quality. The whole genome sequence of pear has also been published with high quality assembly. Therefore, a good platform is available to study the genetic characteristics of fruit development and quality conformation in pear. The transcriptome of Japanese pears has been analyzed to reveal the flower bud transitioning through endodormancy^23^, and occidental pear^24^ was analyzed to study fruit size. However, many fruit developmental characters and transcriptome-based differences in all five pear species are still unclear. RNA-seq technology can be an effective method to reveal gene expression patterns and difference during fruit development and maturation for distinguished cultivated species of pear.

In this research, we performed large-scale RNA sequencing from seven fruit developmental stages in the five cultivated species of pear to effectively reveal the main differences between fruit-related metabolism, especially fruit post- or non post-ripening. We used the ‘Dangshansuli’ (*Pyrus bretschneideri* Rehd.) genome as a reference to analyze various fruit developmental patterns and differentially expressed genes, which mainly regulate the pear growth and fruit quality. Results contribute to a better understanding of the dynamic metabolism and molecular mechanism of different pear species, which provides a significant basis to improve fruit quality of pear, and even other species in Rosaceae.

## Results and Discussion

### RNA sequencing of different developmental stages of pear fruit in five cultivars

In this study, the transcriptomes of five representative cultivars: ‘Hosui’ (*P. pyrifolia*), ‘Yali’ (*P. bretschneideri*), ‘Kuerlexiangli’ (*P. sinkiangensis*), ‘Nanguoli’ (*P. ussuriensis*) and ‘Starkrimson’ (*P. communis*), which are the main cultivated species worldwide, were selected to reveal gene expression patterns and explore relationships between different fruit traits. A total of seven periods (date details in materials), including six key fruit developmental stages (periods 1–6) and one fruit senescence stage after harvesting (period 7) were analyzed. Fruit developmental stages were: fruit setting (period 1), physiological fruit dropping (period 2), fruit rapid enlargement (period 3), a month after fruit enlargement (period 4), pre-mature (period 5) and mature (period 6). Each was sequenced by the Illumina2000 platform. There were more than 10 M raw reads for each sample analysis (Table 1). The original data are available on NCBI: project number PRJNA309745, SRA trace number SRP070620. The mean quality score was above 30, and after adapter removing and ribosomal RNA filtering, a total of 33,136 mapping genes were aligned to the reference pear (*Pyrus bretschneideri* Rehd.) genome^18^, and analyzed based on the 49 bp RNA-seq single end data. The mapping gene numbers were 28,621 (86.4%,‘Hosui’), 29,041 (87.6%, ‘Kuerlexiangli’), 28,925 (87.3%, ‘Nanguoli’), 29,294 (88.4%, ‘Starkrimson’) and 28,905 (87.2%, ‘Yali’). Transcript sets of five cultivars had no bias in mapping gene number, which reflected good sequencing distribution. Little difference was found in the percentage of aligned gene numbers accounting for clean reads during fruit developmental stages, however, it was relatively low for all cultivars at maturation or senescence (Table 1). One major reason might be that many genes have no expression or greatly decreased expression during fruit maturation. Another natural reason could be low sequencing depth, and the limited sequencing data, 10 M reads with 49 bp length for each sample. In tomato, as the cell wall decomposed during maturation, the expression of many genes involved in cell wall carbohydrates also decreased^25^. The same phenomenon was also present in watermelon, with *CmXTH* expression down during mature-fruit abscission^26^.

### Transcriptome sequencing revealed divergent gene expression patterns in pear fruit

**Figure 1:**
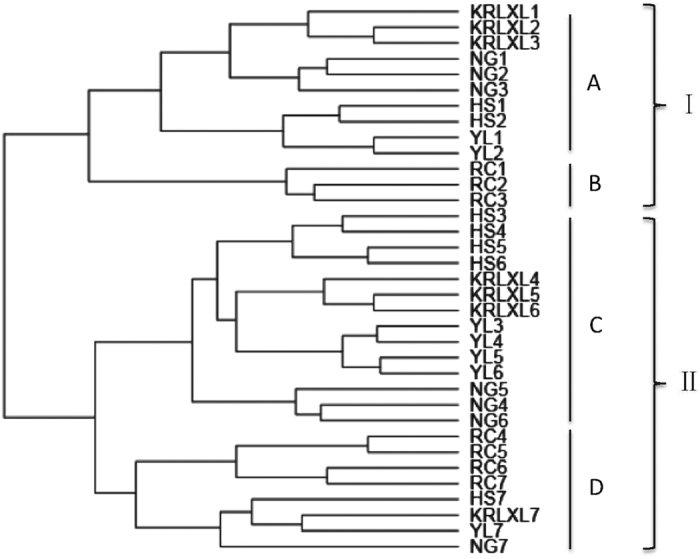
Cluster tree of five pear cultivars during fruit development and maturation stages. HS: Hosui, YL: Yali, KRLXL: Kuerlexiangli, NG: Nanguo, RC: Red Clapp’s Favorite mutant (‘Starkrimson’), 1–7: seven fruit developmental stages. Spearman correlation tree has two major groups, I and II and four subgroups, A–D.

Regular mRNA abundance was calculated by reads per exon kilobase per million mapped sequence reads (RPKM). Cluster 2.0^27^ used clustering correlation matrixes to generate a tree. As Fig. 1 shows, there were two main groups in the tree, group I and group II. Most fruit setting stages (period 1) and fruit physiological dropping stages (period 2) were gathered together in group I by the Spearman correlation method. The occidental pear ‘Starkrimson’ made up a separate subgroup B, and other oriental pears made subgroup A. Group II also divided into two subgroups, C and D, with subgroup C clustering the stages of full development to mature stage of all oriental cultivars, while subgroup D included post-harvest stages of all five cultivars and three developmental stages of occidental pear ‘Starkrimson’. It was interesting to find that the most closely related stages of the same cultivar usually first clustered together, and then clustered with stages of other cultivars, indicating different gene expression patterns between the five cultivars, which represent the five cultivated species to some extent. In addition, occidental pear ‘Starkrimson’ showed a relatively distant relationship with other oriental cultivars, in both groups I and II.

**Figure 2:**
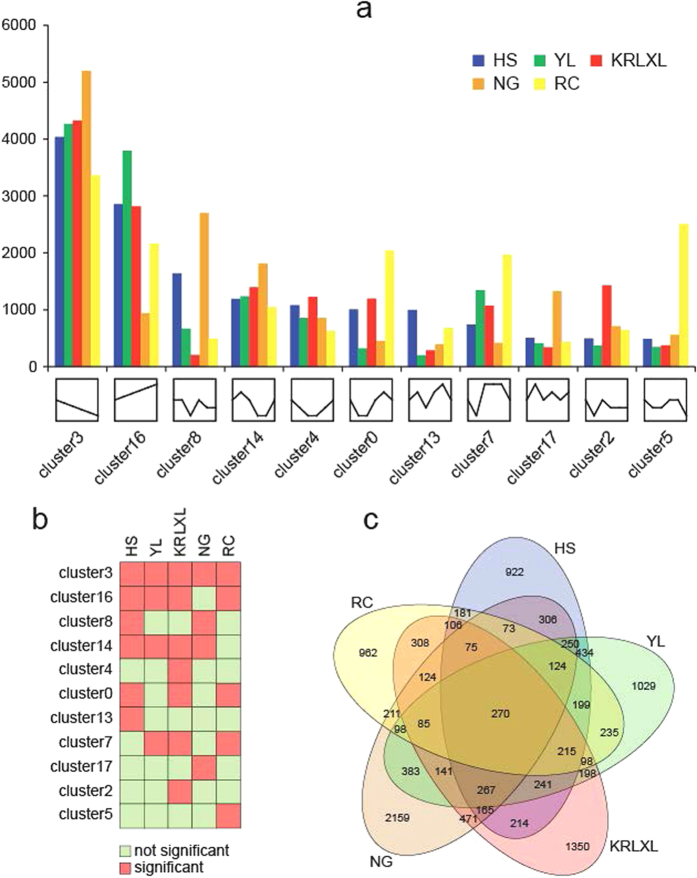
Gene expression clusters for different Pyrus species. (**a**) Main clusters of gene expression pattern of five Pyrus species. X-axis: type of main clusters and Y-axis: number of genes in different clusters. (**b**) Significance level of gene number in different clusters for five species. (**c**) Venn diagram for gene number in the continuous decrease pattern (cluster 3).

**Figure 3:**
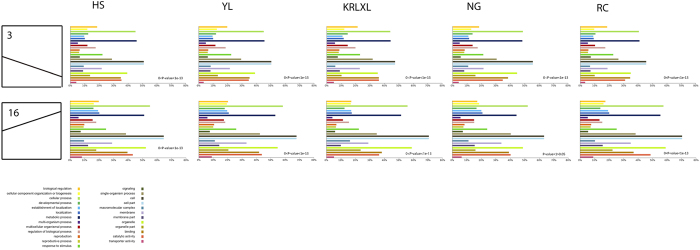
Common and different gene expression clusters and their function in five cultivars. Two main gene expression patterns are illustrated on the left, namely cluster 3 and cluster 16. Functional analysis based on the five cultivars is presented in different colors.

Models for genes with the same expression pattern were defined by STEM analysis^28^, and a total of 20 gene cluster modules were generated for each sample. We selected the top eleven with the same expression model, shown in Fig. 2a. In general, the gene expression models cluster 3–steady decrease–and cluster 16–steady increase–represented the most common gene expression models across all seven stages and different cultivars. Cluster 3 included the largest number of genes (more than 3,000 genes for each cultivar), indicating that all five cultivars had most genes expressed with the model of steady decrease. A total of 270 genes were identified in all five cultivars in cluster 3, demonstrated in the Venn diagram (Fig. 2c). Gene expression modules of five pear cultivars were significantly enriched (P-value < 1e-5, except for ‘Nanguo’ in cluster 16) in two main clusters, and presented different genetic functions by Gene Ontology (GO) (Fig. 3). Continuous increase cluster 16 had a relatively higher percentage of genes responsible for cellular component organization or biogenesis, single–organism process, cell part and transporter activity, compared with the steady decrease cluster 3 (Fig. 3). We used the 270 genes in cluster 3 for KEGG pathway enrichment, and the genes were most enriched in the photosynthesis pathway (map00195 with p-value of 4.15e-12 and map00196 with p-value of 5.68e-6). In previous gene expression pattern research, sweet orange showed the largest cluster (3,075 genes, accounting for 16.3%) expressed as continuous increase during four fruit development and ripening stages, and the genes in that cluster had functions such as cysteine proteinase sucrose phosphate synthase, and a sucrose transporter^29^. However, the steadily changing gene-expression patterns were not the main models^22^ in studies of the berry transcriptome, based on analysis of three stages of fruit development.

Specific gene expression patterns showed that occidental pears had a gradually decreasing tendency of gene expression in stage 6 of ‘Starkrimson’ – a typically European pear species, expressed genes mostly grouped in cluster 3, cluster 16, cluster 0, cluster 7, and cluster 5 (Fig. 2b). All the gene expression tendencies in these clusters were down-regulated at the mature stage, except for the common model of cluster 16. Cluster 2 represented a specific gene expression pattern of ‘Kuerlexiangli’, because the gene numbers in that pattern were significantly high (under the significance level of 0.05, Fig. 2b). In addition, ‘Nanguoli’ showed its own special fruit developmental model with cluster 17, and ‘Hosui’ had a special gene expression cluster 13. How these specific gene expression patterns regulate the particular fruit development of each cultivar still needs further exploration and study.

### Specific gene expression characteristics for post-ripening and non post-ripening cultivars in pear

**Figure 4:**
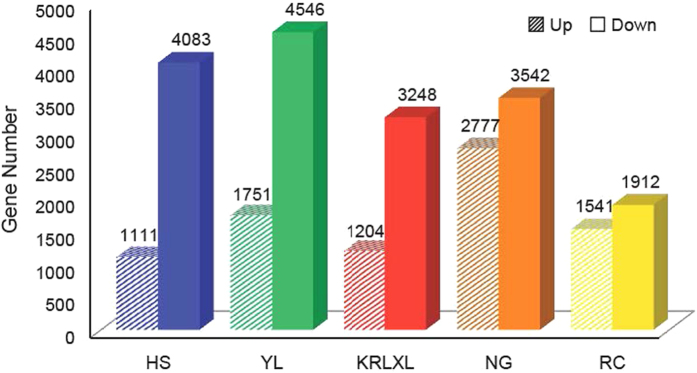
Up- and down-regulated genes from maturity to post harvest in pear species. X-axis indicates five samples, and Y-axis is gene number from maturity to post harvest. Bars with diagonal lines are up-regulated gene numbers, and solid bars are down-regulated gene numbers.

We sequenced samples from the stage of fruit maturity to senescence to detect maturation characteristics of pear. Of the five cultivated species, ‘Starkrimson’ and ‘Nanguoli’ are post-ripening pears, while other three cultivars, ‘Hosui’, ‘Yali’ and ‘Kuerlexiangli’ are eatable directly after harvest. Analysis revealed that post-ripening cultivars were still vigorous after harvest, furthermore we found that they had a higher proportion of up-regulated genes. ‘Nanguoli’ had 43.95% (Fig. 4) up-regulated genes from maturity to fruit senescence. The same tendency was followed by another post-ripening occidental pear, ‘Starkrimson’, with 44.63% (Fig. 4). However, up-expressed genes in ‘Hosui’, ‘Yali’, and ‘Kuerlexiangli’ only accounted for 21.39%, 24.33%, and 27.04% (Fig. 4), respectively. The ratio of up-expressed genes between post-ripening and non post-ripening pear cultivars had significant differences by t-test (p-value = 0.005032).

**Figure 5:**
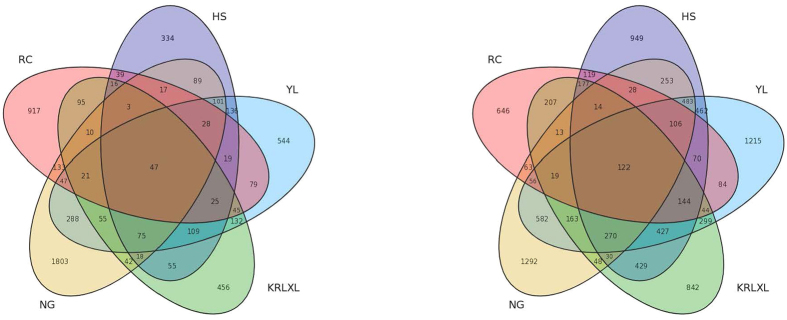
Venn diagram of up- and down-regulated genes.

In order to distinguish the two extreme gene expression patterns in post-ripening and non post-ripening pears, we explored gene functions within the two patterns. The number of genes only up-expressed in post-ripening cultivars was 133, while the gene number of only down-expressed in non post-ripening cultivars was 47, as Venn diagrams in Fig. 5 show for the up-regulated and down-regulated genes. Previous studies proved that ethylene plays an important role in catalyzing ripening in European pears^30^. Ethylene catalyzed the maturity process through different methods such as with the involvment of one *cupin family protein* gene and two unannotated genes in occidental pears^10^. In this study, functional analysis showed that the only up-regulated genes in post-ripening cultivars were responsible for nucleosome assembly and protein-DNA complex assembly based on the gene ontology (GO) analysis. However, the only down-expressed genes in non post-ripening cultivars were in the categories metabolism and transportation.

### Gene expression differences reveal zeatin and ethylene regulating development and maturation of pear fruit

Zeatin is a plant hormone, first isolated from maize. It controls many aspects of plant development, such as cell division^31^ and regulation of environmental stress responses^32^. Based on the heat map of gene expression at later stages of fruit development, we found cytokinin oxidize (CKX) to be differentially expressed (with log2 fold change from 4 to 8) in all five species (Fig. S1). *CKX* is the key gene for zeatin synthesis, and is highly conserved in function and structure. Not only did the gene expression pattern of *CKX* seem to be the same among different pear cultivars (Fig. S1), but also the structure of CKX was conserved between families^33^.

Ethylene regulates fruit maturity and also responses to stress in plants^34^. We selected rate-limiting genes in ethylene biosynthesis and key genes for ethylene receptors, though maturity characteristics regulated by ethylene varied between different pear cultivars. Genes corresponding to ethylene biosynthesis had two expressed forms, one pattern was steady increase, and the other pattern steady decreased during fruit development. S-adomet synthetase (SAMS), 1-aminocyclo-propane-1-carboxylic acid synthase (ACS) and 1-aminocyclo-propane -1-carboxylic acid oxidase (ACO) were rate-limiting for ethylene synthesis. Expression of *Pbr018549.1*, *Pbr037756.1*, *Pbr0001686.1*, and *Pbr008602.1* (Fig. S2) was continually high for SAMS, but low for *Pbr020754.1*, *Pbr026061.1*, *Pbr006707.1* and *Pbr018549.1* in five cultivars. The expression of two *ACS* related genes (*Pbr030234.1* and *Pbr029891.1*) was especially high at the stage of fruit maturation. The oriental post-ripening cultivar ‘Nanguoli’ also showed a high expression of *ACS* at the mature stage (period 6) and fruit senescence stage (period 7), and the same result was verified in previous studies^35^. In *Pyrus pyrifolia*, ethylene receptors and *ACS* have also been identified as regulating pear maturation in the late developmental stages^36^. We found that most ethylene receptors had steady expression. Ethylene response (*ETR*), constitutive triple response (*CTR*) and ethylene insensitive (*EIL*) are the main ethylene receptors; a heat map of these gene expression was shown in Fig. S2. In addition, RAN (response to antagonist) is a copper transporter necessary for ethylene signaling^37^. In our study, RAN (*Pbr002476.1*) was shown to express especially highly at the early stage of all five pear cultivars (Fig. S2).

### Gene expression reveals genetic differences related to fruit quality in pear

#### Stone cell

The stone cell is a special characteristic of pear fruit that can considerably influence fruit quality. Lignin has been found to be the main component of stone cells^38^. So, in our study, lignin synthesis-related genes were selected for gene expression analysis (Fig. S3). The results showed that several genes such as *4CL* (4-coumarate CoA ligase), *C3H* (*p*-coumarate 3-hydroxylase), *CA5H* (coniferylaldehyde 5-hydroxylas), and *CAD* (cinnamyl alcohol dehydrogenase) had relatively high expression at the early fruit development for all five cultivars (Fig. S3), which was consistent with previous studies showing that stone cells could be detected from sixty days after full blooming^39^. Genes regulating hydroxycinnamoyl transferases (HCT), which reduce the H-lignin content^40^, were identified as expressed at the early stage of fruit development (Fig. S3). Evaluation of stone cell content in 304 pear cultivars from five species revealed that the mean stone cell content in *P. ussuriensis* was the highest^4^. In this study, it was found that Caffeoyl-CoA *o*-methyltransferase (CCOMT) related gene (*Pbr034039.1*) expressed at an especially low level during ‘Nanguoli’ development, with higher expression in the other four cultivars. Based on the lignin pathway analysis from previous study, *CCOMT* uses the lignin precursor to synthesize G-lignin and S-lignin^41^. Therefore, we speculated that ‘Nanguoli’ might have relatively high H-type lignin.

#### Sugar and acid biosynthesis

Sugar and acid are among the most important fruit quality indicators, and the ratio of sugar/acid is a large component of fruit flavor. Sugars are not only energy for metabolism, but also signaling materials for plants. Fructose accounts for the greatest sugar content at maturity for the five pear cultivars, from components of sucrose, glucose, fructose, and sorbitol (Fig. S4). Among these cultivars, ‘Starkrimson’ had high fructose content, exceeding others at the mature stage (Fig. S4). Meanwhile, we found that expression of one hexokinase (*HK*) related gene in ‘Starkrimson’ was not as high as in the other four species (Fig. S5, *Pbr005841.1*). Since hexokinase can transfer fructose into fructose-6-phosphate, we speculated that low *HK* expression in ‘Starkrimson’ induced higher fructose content. Similarly, *HK* was also able to induce fructose repression in yeast^42^.

Organic acid is an important factor that can affect fruit flavor. Malate and citrate are the main acids in pear fruit. From malate, citrate, quinate and shikimic acid content at the mature stage, citrate in ‘Starkrimson’ was the highest among all the acids of five species^43^. It was found that citrate synthase related genes (*CS*, *Pbr004985.1*) always had superior expression in ‘Starkrimson’ during fruit development, and could be an important candidate gene for controlling acid content. In a previous report, two Chinese pear accessions were analyzed to find that *CS* also responds for citrate accumulation^44^.

### Gene networks reflect ethylene synthesis and ethylene receptor interaction

**Figure 6:**
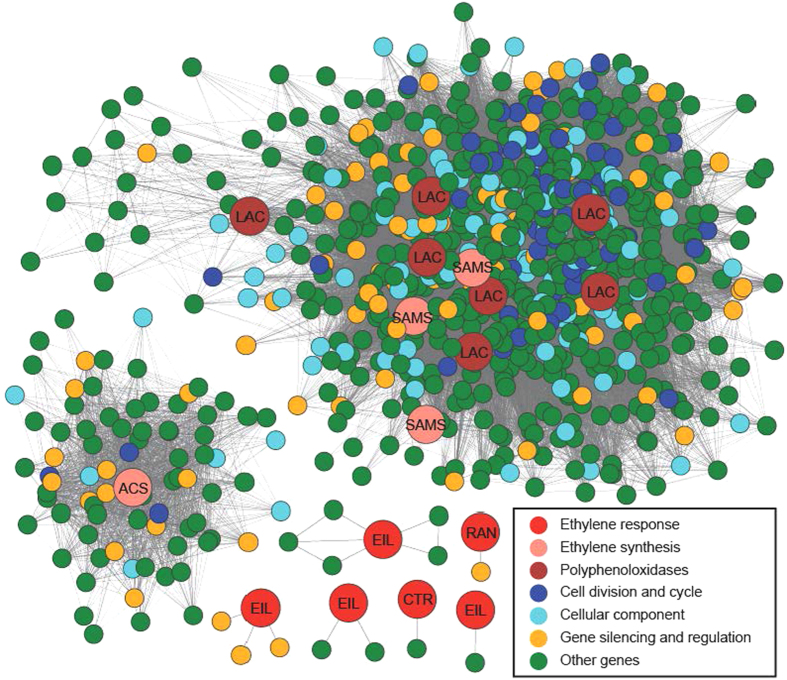
Network of ethylene-related genes for pears. ACS: 1-aminocyclo-propane-1-carboxylic acid synthase; SAMS: S-adomet synthetase; LAC: laccase.

In order to understand which gene networks are active in pear fruit development, we explored the ethylene synthesis and ethylene receptor interaction with the R package. Based on the results presented in Fig. 6, we found that several ethylene synthesis genes and polyphenoloxidase-related genes interacted with each other directly, and indirect relation was reflected between some ethylene synthesis genes and ethylene response genes. Gene interaction analysis illustrated that *SAMS* (S-adomet synthetase) regulated ethylene synthesis together with *LAC* (laccase), which had a tight relationship during pear fruit development. In addition, genes controlling cell division, cellular components, gene silencing, and regulation functioned in a network with indirect complex interaction (Fig. 6). It was reported that S-adomet synthetase (SAMS) can synthesize S-adenosyl-methionine (S-AdoMet) from methionine with ATP^45^. The laccase-related gene, as an ethylene receptor, regulates the downstream ethylene signal transduction pathway^46^. Researchers found that a cDNA induced by ethylene was homologous to a *LAC* gene in Rosaceae species of miniature roses^47^, which was also the verification of the close connection of ethylene and *LAC*.

### SNP mining for expressed genes in fruit from five different pear cultivars

#### SNP calling based on transcriptome

**Figure 7:**
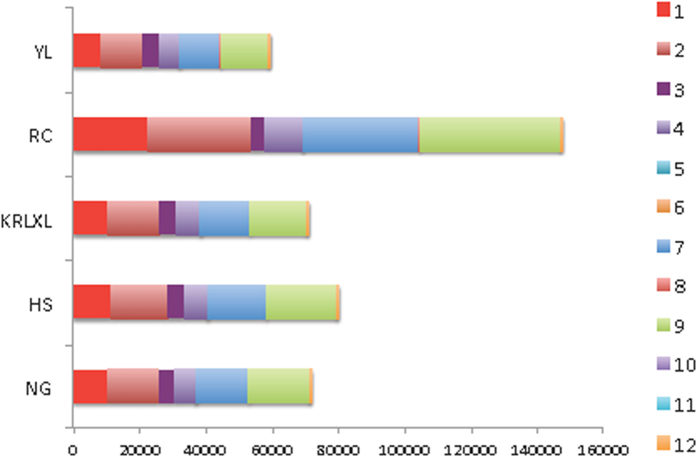
SNP number and effectiveness in five pear species. X-axis presents SNP effectiveness number, and Y-axis shows five pear species. SNP effectiveness types: 1 SNPs are present in the upstream region of 2 Kb. 2 SNPs are present in the downstream region of 2 Kb. 3 SNPs are present 2 bp around the boundaries of exon and intron. 4 SNPs are in introns caused by selective splicing. 5 SNPs appear in the initiation codon and change the function. 6 SNPs appear in the termination codon and change the function. 7 SNPs appear in the exon and cause synonymous mutation. 8 SNPs are present in the exon and cause premature stop codons. 9 SNPs appear in the exon and cause nonsynonymous mutation. 10 SNPs appear in the termination codon and do not change the function. 11 SNPs appear in the initiation codon and do not change the function. 12 SNPs meet the missing ‘N’ in the genome.

SNPs were detected in ‘Hosui’, ‘Yali’, ‘Kuerlexiangli’, ‘Nanguoli’, and ‘Starkrimson’, based on the RNA-seq data during fruit development in comparison with 'Dangshansuli’ (*Pyrus bretschneideri*) genome^18^. SNP calling based on the transcriptome is meaningful, because some SNPs can affect protein function. Oriental pears ‘Hosui’, ‘Yali’, ‘Kuerlexiangli’, and ‘Nanguoli’ had 83543, 62522, 74814 and 75463 filtered SNPs, respectively. We found the most polymorphic cultivar was ‘Starkrimson’, with 154,404 SNPs after filtering (Fig. 7). The nearly doubly high diversity rate represented the many differences between oriental pear ‘Dangshansuli’ and occidental pear of ‘Starkrimson’ on the transcriptome level.

#### Effectiveness of non-synonymous SNPs

In previous reports, SNPs induced mutants for gene function, for example, peach genotype of flesh melting was affected by SNPs at the melting flesh (M) locus^48^, and SNP241 in watermelon resulted in a resistant gene mutation on exon^49^. Arrays of apple^50^ and peach^51^ have also been developed based on SNPs, and could be used for genetic mapping, genetic relatedness and marker assisted selection in breeding programs^52^. In our research, SNPs were classified by the location and effectiveness in genes. Effectiveness included synonymous mutations, non-synonymous mutations, start codon mutations and stop codon mutations, among others, the proportion varied in species (in Fig. 7). Synonymous mutations were overwhelmingly prevalent over other types in all five cultivars. Non-synonymous SNPs in the initiation codon (type a, eg: HS accounted for 0.015% in 79,724 SNPs), SNPs in the termination codon (type b, eg: HS: 0.039%), and SNPs changing protein function by early termination (type d, eg: HS: 0.05%) accounted for only a small portion in the whole SNPs (in Table 2 and Fig. 7), however, these SNPs sometimes can completely change gene function and effect the biology trait significantly.

Based on the gene ontology of the predicted SNP-induced varied genes in Table 2, we found that genes responsible for transport in ‘Nanguo’ had the largest number of mutations in the start codon regions, and GO slim term of response to stress was prone to mutate in the stop codons comparing with other GO terms. We demonstrated all the SNPs, which induced mutations in start codon, stop codon and premature stop codon in Table S1. Conserved function domains (https://www.ncbi.nlm.nih.gov/cdd) were analyzed based on these genes (Table S2). Many SNPs are related to fruit development. We found a SNP mutation in the start codon of *Pbr038562.1*, which had the functional domain of “ethylene responsive element binding protein (EREBP)” in cultivar of ‘Starkrimson’. In Arabidopsis, EREBPs can interact with the 11 bp GCC box in the promoter of target genes and activate downstream ethylene responses^53^. Furthermore, in the stop codon region, the SNP from ‘Kuerlexiangli’, ‘Nanguo’ and ‘Starkrimson’ might influence *Pbr026622.1* with domains from the DUF647 superfamily that has functions in auxin transport. A study found that a member of the conserved DUF647 protein family plays an essential role in the regulation of polar auxin transport by maintaining the proper level of auxin transporters on the plasma membrane^54^. The predicted non-synonymous SNPs provided valuable information for candidate genes associated with fruit development, even though more gene function experiment should be performed to verify the SNP effectiveness derived gene mutations.

### RNA expression verification by q-PCR on ‘Starkrimson’

**Figure 8:**
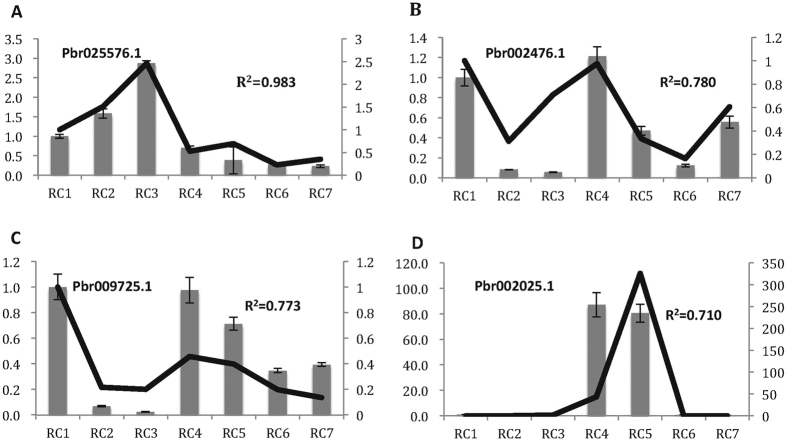
Comparison of RNA-seq based gene expression and q-PCR verification. Figures represent expression of four genes for seven fruit developmental stages of ‘Red Clapp’s Favorite’. X-axis represents the fruit developmental stages. Y-axis on the left shows the relative gene expression levels of q-PCR by bar charts, and the Y-axis on right illustrated the relative values of RPKM with line charts. The RPKM of Y-axis on the right were unified by dividing the first stage’s gene expression value in (**A–C**).

We selected five genes to verify the RNA-seq gene expression data from seven stages of fruit development for ‘Starkrimson’ by q-PCR. Each gene was repeated in three biological samples with the first period’s expression as a standard level. Data for the genes β-amyrin synthesis (*Pbr025576.1*), ethylene receptor (*Pbr002476.1*), ABA synthesis (*Pbr009725.1*), and zeatin synthesis (*Pbr002025.1*) are shown in Fig. 8. Correlations of the RPKM of four genes and the relative qPCR expression were 0.983 (*Pbr025576.1*), 0.780 (*Pbr002476.1*), 0.773 (*Pbr009725.1*), and 0.710 (*Pbr002025.1*), throughout fruit development, respectively. The ethylene receptor showed fluctuating expression. We found that the ABA synthesis related-gene had high expression at the early stage of fruit development, when xanthoxin is being converted to ABA^55^. The zeatin-related gene was found to have a peak in the middle stage of fruit metabolism in ‘Starkrimson’, but was highly expressed in the early stage of fruit development in blueberry^56^.

## Methods

### Pear Cultivar selection

Cultivars of ‘Hosui’, ‘Yali’, ‘Kuerlexiangli’, ‘Nanguoli’ and ‘Starkrimson’ originate from different species, and have a range of maturity characteristics. We collected pear fruits from the Jiangpu Farm of Nanjing Agricultural University. A total of seven developmental stages were collected from the fruit-setting period, which was 15 days after full blooming (15 DAB, period 1). Pear fruits were selected from the physiological fruit dropping stage (30 DAB, period 2), fruit rapid enlargement stage (55 DAB, period 3), a month after fruit enlargement stage (85 DAB, period 4), pre-mature stage (115 DAB, period 5), mature stage (varied according to species, period 6) and fruit senescence stage (period 7). Then pear fruit flesh was cut into small pieces and stored in the freezer at −80 °C.

### RNA sequencing and sequence mapping

A total of 35 samples’ (five species X seven stages) RNA was extracted from fruit flesh. Illumina standard mRNA-Seq Prep Kit (TruSeq RNA and DNA Sample Preparation Kits version 2) was used for construction of RNA sequencing libraries. Single end RNA-seq data were generated with length of 49 bp. Reads were filtered and trimmed, and then they were mapped onto ‘Dangshansuli’ (*Pyrus bretschneideri*) CDS sequences by software of SOAPaligner^57^.

### Gene expression and clustering analysis

The gene expression profile was presented by in-house perl scripts using RPKM algorithm^58^. The Java-based program STEM^28^ was used for clustering to build gene expression model. All of the mapping units with at least five expression sets were used to map onto the gene model.

### Gene co-expression analysis

WGCNA (weighted gene co-expression network analysis) package^59^ was used to find clusters (modules) of highly correlated genes and construct co-expression networks. Then Cytoscape (http://www.cytoscape.org) was used to visualize the correlation relationships between specified genes.

### SNP calling

Burrows-Wheeler Alignment (BWA)^60^ was used for RNA-seq read mapping (-n 1 -o 1 -e 0). After files were aligned for all 35 samples, bam format files were merged together from seven stages of the same cultivar by samtools^61^. SNP calling was performed with the module ‘mpileup’ in samtools. Effectiveness of the SNPs was analyzed with an in-house perl script (https://github.com/Sunhh/NGS_data_processing).

### Sugar content measurements

Pear fruit sugars including sucrose, glucose, fructose and sorbitol were measured using High Performance Liquid Chromatography (HPLC). Sugars were extracted from pear flesh by grounding, and then were dissolved and filtered through a SEP-C18 cartridge (Waters, WAT021515) and Sep-Pak filter. Waters 1525 system (Waters, Shanghai, China) was used to measure sugars. The column was 6.5 mm × 300 mm, Inner Diameter, 10 um particle size (Waters), with a guard column cartridge of Sugar-pak 1 Guard-Pak Holder and Insert (Waters). Column temperature was 85 °C, and the reference cell temperature was 35 °C.

### RT-PCR validation

Several expressed genes were selected for verification by quantitative RT-PCR (qRT-PCR) on ‘Starkrimson’. Coding sequences of the genes were acquired from the pear genome project (http://peargenome.njau.edu.cn). Primers for the candidate genes were designed (Table S1) with the online software Primer 3 (http://simgene.com/Primer3). The LightCycler 480 SYBR GREEN Master (Roche, USA) system was used, with *GAPDH* as the house-keeping gene. Reactions of target genes and reference genes were performed in triplicate in a total volume of 20 μl. PCR reactions were carried out via an initial incubation at 95 °C for 10 min and at 95 °C for 15 s, and then cycled at 60 °C for 15 s, and 72 °C for 20 s for 40 cycles, and final extension at 72 °C for 30 min.

## Additional Information

**How to cite this article**: Zhang, M.-Y. *et al*. Distinct transcriptome profiles reveal gene expression patterns during fruit development and maturation in five main cultivated species of pear (*Pyrus L.*). *Sci. Rep.* **6**, 28130; doi: 10.1038/srep28130 (2016).

## Supplementary Material

Supplementary Data

Supplementary Figures

## Figures and Tables

**Table 1:** Read number and unique gene mapping for RNA-seq.

Samples	No. of raw reads	No. of clean reads	No. of mapping genes	Percentage of clean read mapping rates (%)
HS1	11,302,851	10,328,076	25,688	75.63
HS2	10,154,316	9,197,441	24,765	75.11
HS3	12,117,316	9,398,656	23,071	74.06
HS4	11,213,396	10,109,015	23,635	74.63
HS5	11,243,703	10,216,762	22,655	75.76
HS6	11,950,723	11,000,668	22,663	75.42
HS7	10,668,693	9,779,232	21,374	73.93
KRLXL1	12,362,986	11,781,418	26,289	73.72
KRLXL2	12,239,312	11,619,212	24,464	73.25
KRLXL3	11,970,718	11,341,031	24,826	73.18
KRLXL4	12,395,731	11,272,539	23,929	73.38
KRLXL5	11,885,029	10,253,055	23,148	72.44
KRLXL6	12,197,320	11,152,288	23,282	73.74
KRLXL7	11,847,303	11,274,519	22,249	73.20
NG1	12,516,088	12,006,578	26,173	74.42
NG2	11,942,855	11,447,523	25,681	74.55
NG3	11,548,878	9,242,452	23,346	75.35
NG4	12,396,607	10,214,122	23,310	75.36
NG5	12,533,756	8,731,570	21,469	75.09
NG6	12,428,560	11,425,645	22,259	74.98
NG7	12,461,123	11,408,415	21,801	74.97
RC1	11,854,995	11,287,205	25,675	73.62
RC2	11,695,092	11,085,589	25,046	73.10
RC3	12,087,344	11,566,921	24,728	71.81
RC4	11,734,817	11,298,495	24,419	73.45
RC5	11,678,523	11,180,375	24,539	72.98
RC6	11,693,653	11,288,167	22,323	74.25
RC7	12,468,584	11,734,107	22,473	76.01
YL1	12,098,295	11,396,714	26,062	75.83
YL2	12,449,966	11,712,248	25,643	75.47
YL3	12,564,888	11,788,331	24,220	73.63
YL4	12,257,369	10,177,818	23,495	73.74
YL5	12,304,901	10,475,211	23,431	74.09
YL6	11,932,440	11,371,346	22,837	74.24
YL7	11,123,512	10,582,639	21,394	75.92

**Table 2:** GO slim gene counts in five pear cultivars.

GO Slim Term	HS				KRLXL				NG				RC				YL			
	a	b	c	d	a	b	c	d	a	b	c	d	a	b	c	d	a	b	c	d
metabolic process	3	8	2427	8	3	11	2304	9	0	14	2422	9	4	17	3961	9	1	9	1928	7
biological process	3	10	2178	9	2	8	2004	5	3	10	2105	9	8	14	3342	20	2	11	1657	8
cellular process	4	8	2109	8	2	9	1992	6	2	13	2044	7	3	15	3404	7	1	10	1656	6
protein metabolic process	2	3	932	4	1	4	842	5	0	5	926	2	1	3	1437	4	1	4	729	3
biosynthetic process	1	4	730	2	1	5	711	2	0	5	725	3	2	6	1240	0	0	5	578	5
nucleobase-containing compound metabolic process	0	2	583	3	0	1	563	1	0	4	567	1	1	5	977	0	0	1	446	2
cellular protein modification process	1	1	557	2	0	2	495	1	0	3	554	0	0	2	866	3	0	2	426	0
transport	1	1	413	1	0	0	443	1	3	1	426	2	0	1	702	4	0	1	361	1
carbohydrate metabolic process	0	0	250	1	0	0	211	0	0	0	235	2	0	2	377	0	0	0	191	0
response to stress	1	3	222	2	0	4	209	0	0	1	213	1	0	3	322	1	0	2	200	0
lipid metabolic process	1	0	166	0	1	2	148	0	0	1	158	1	1	1	249	1	1	1	112	0
catabolic process	0	0	147	1	0	1	129	0	0	0	141	1	0	2	223	1	0	0	117	0
cellular component organization	0	0	144	1	0	0	134	0	0	1	130	1	0	0	208	1	0	0	111	0
translation	0	2	128	0	0	2	117	2	0	2	123	1	0	1	189	0	0	2	85	2
cell communication	1	1	122	1	0	1	122	1	0	1	125	0	0	3	216	1	0	2	109	0
signal transduction	1	1	108	0	0	1	110	0	0	1	105	0	0	3	184	0	0	2	93	0
DNA metabolic process	0	0	81	1	0	0	79	0	0	1	73	0	0	0	115	0	0	0	63	0
generation of precursor metabolites and energy	0	0	53	0	0	0	36	0	0	0	47	0	0	1	77	0	0	0	38	0
response to biotic stimulus	0	1	13	0	0	1	6	0	0	1	8	0	0	1	14	0	0	1	7	0
pollination	0	0	13	1	0	0	11	1	0	0	19	0	0	0	31	1	0	0	15	0
pollen-pistil interaction	0	0	13	1	0	0	11	1	0	0	19	0	0	0	31	1	0	0	15	0

a: SNPs appear in the initiation codon and change the amino acid function; b: SNPs appear in the termination codon and change the amino acid function; c: SNPs appear in the exon and cause amino acid non-synonymous mutation; d: SNPs appear before the termination codon and change the amino acid function by early termination.
