# Supplementary material for: Distinct transcriptome profiles reveal gene expression patterns during fruit development and maturation in five main cultivated species of pear (Pyrus L.)
Source: Sci Rep. 2016 Jun 16;6:28130. doi: 10.1038/srep28130 (PMC4910100; doi:10.1038/srep28130)
Supplement: Supplementary Figures [file srep28130-s2.doc]

**Distinct transcriptome profiles reveal gene expression patterns during fruit development and maturation in five main cultivated species of pear (*Pyrus L.*)**

Ming-Yue Zhang^1^, Cheng Xue^1^, Linlin Xu^1^, Honghe Sun^2^, Meng-Fan Qin^1^, Shaoling Zhang^1^, Jun Wu*^1^

^1^ Centre of Pear Engineering Technology Research, State Key Laboratory of Crop Genetics and Germplasm Enhancement, Nanjing Agricultural University, Nanjing 210095, China

^2^ Beijing Academy of Agriculture and Forestry Sciences, Key Laboratory of Biology and Genetic Improvement of Horticultural Crops (North China),
Beijing, 100097, China

*Corresponding author: Jun Wu

E-mail: [wujun@njau.edu.cn](mailto:wujun@njau.edu.cn)

Tel: +86-(025)84396485

Fax: (025)84396485

**Figure S1 Key genes expression for zeatin synthesis**

IPT: cytokinin synthase; CYP735A: cytokinin trans-hydroxylase; CKX: Cytokinin dehydrogenase; UGT76C1_2: cytokinin-N-glucosyltransferase; TRIT1: tRNA dimethylallyltransferase; CISZOG: cis-zeatin O-glucosyltransferase.


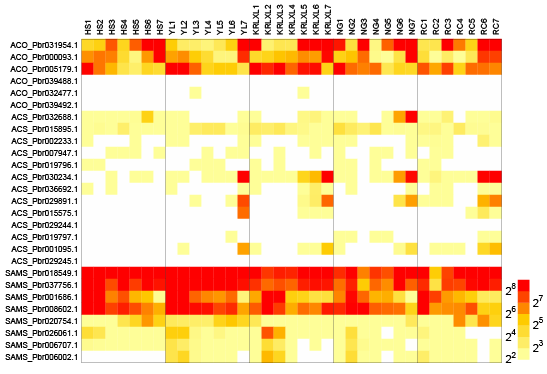

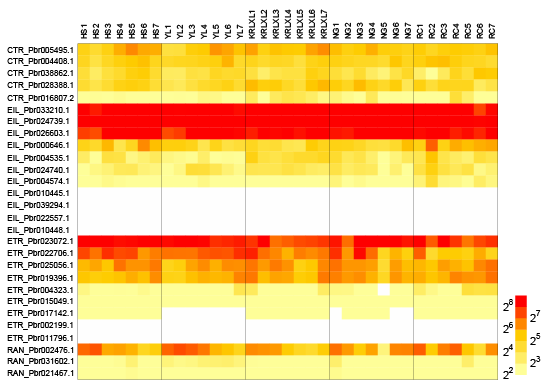


**Figure S2 Gene different expression for ethylene biosynthesis and ethylene receptors**

ACO: 1-aminocyclo-propane-1-carboxylic acid oxidase; ACS: 1-aminocyclo-propane-1-carboxylic acid synthase; SAMS: S-adomet synthetase.

CTR: constitutive triple response; EIL: ethylene insensitive; ETR: ethylene response; RAN: response to antagonist.


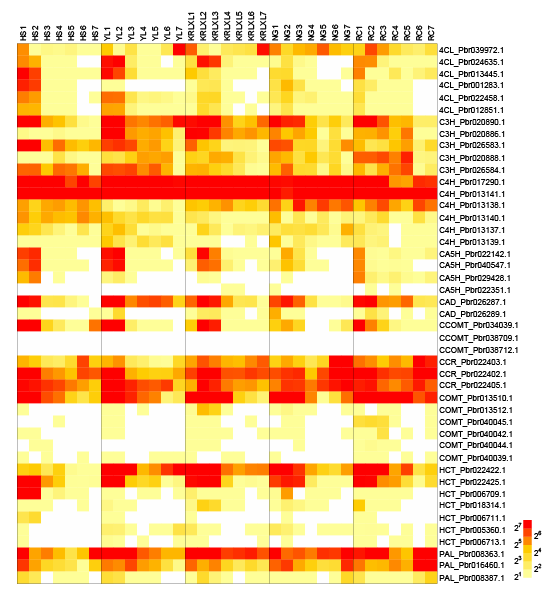


**Figure S3 Heat map of lignin synthesis genes**

4CL: 4-coumarate CoA ligase; C3H: *p*-coumarate 3-hydroxylase; C4H: cinnamate 4-hydroxylase; CA5H: coniferylaldehyde 5-hydroxylas CAD: cinnamyl alcohol dehydrogenase; CCOMT: caffeoyl-CoA *o*-methyltransferase; CCR: cinnamoyl-CoA reductase; COMT: caffeic acid *o*-methyltransferase; HCT: hydroxycinnamoyl transferases; PAL: phenylalanine ammonia-lyase.


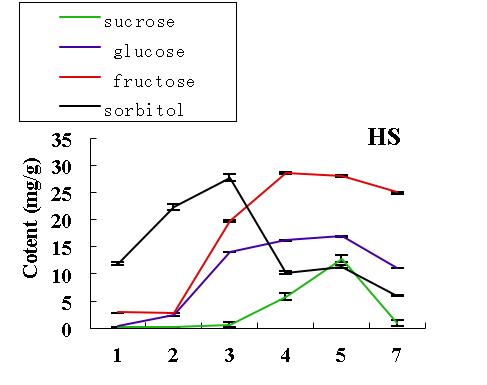

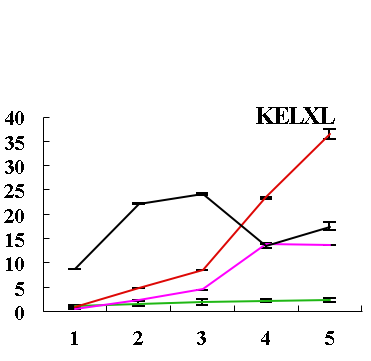


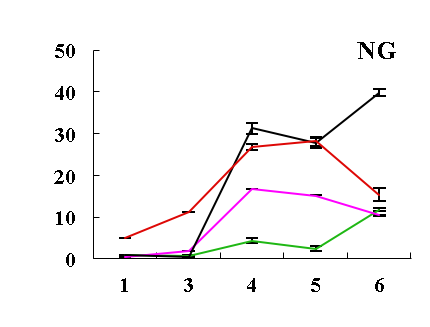

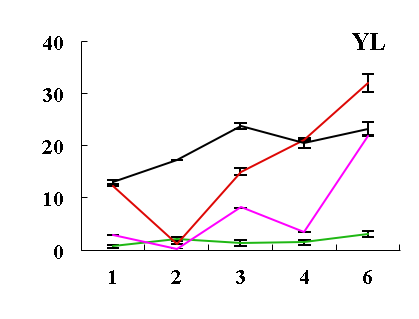


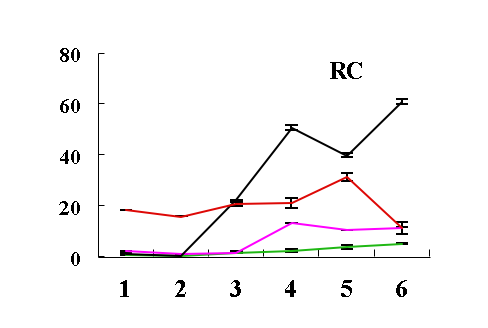


**Figure S4 Sugar content in five pear species by HPLC**

**Figure S5 Heat map of sugar differently expressed genes**

HT: hexose transporter; ALD: acetaldehyde dehydrogenase; F16BP: fructose-1,6-bisphosphate; FK: fructokinase; HK: hexokinase; PFK-ATP: ATP dependent phosphofructokinase; PFP: phosphotransferase; PGI: phosphoglucose isomerase; PGM: phosphoglucomutase; SUT: sucrose transporter.
